# Assessment of LLMs on basic bioinformatics tasks requiring multi-step reasoning and code writing

**DOI:** 10.1093/bioinformatics/btag749

**Published:** 2026-10-30

**Authors:** Varsha Rajesh, Maceo Blumberg, Shreya Jain, Geoffrey H Siwo

**Affiliations:** College of Engineering, University of Michigan, Ann Arbor, MI, 48109-2054, United States; Department of Molecular, Cellular and Developmental Biology, University of Michigan, Ann Arbor, MI, 48109-2054, United States; Department of Learning Health Sciences, University of Michigan Medical School, Ann Arbor, MI, 48109-2054, United States; Department of Learning Health Sciences, University of Michigan Medical School, Ann Arbor, MI, 48109-2054, United States; Department of Pharmacology, University of Michigan Medical School, Ann Arbor, MI, 48109-2054, United States; Center for Global Health Equity, University of Michigan, Ann Arbor, MI, 48109-2054, United States

## Abstract

**Motivation:**

Large Language Models (LLMs), AI agents and co-scientists promise to accelerate scientific discovery across fields ranging from chemistry to biology. Bioinformatics—the analysis of DNA, RNA and protein sequences plays a crucial role in biological research and is especially amenable to AI-driven automation given its computational nature. Here, we assess the bioinformatics capabilities of popular general-purpose LLMs on a set of tasks covering basic analytical questions that include code writing and multi-step reasoning in the domain.

**Results:**

Utilizing questions from Rosalind, a bioinformatics educational platform, we compare the performance of the LLMs on 104 questions relative to expert ground truth and 68 760 humans with varying levels of expertise. Among the frontier LLMs evaluated (GPT-5.2, GPT-4o, GPT-3.5, Llama-3-70B, Llama-4 and Claude Sonnet 4.6), GPT-5.2 exhibited top performance, providing correct answers for 80/104 (77%) questions followed by Claude Sonnet 4.6. LLM performance varied widely depending on the biological categories of questions. While the latest generation of frontier LLM models showed overall improvements in performance, their performance in specific categories of biological questions was lower than first generation models. Overall, LLMs performance mirrored that of humans with lower performance in tasks in which humans had low performance and vice versa. However, LLMs also failed in some instances where most humans were correct and, in a few cases, LLMs excelled where most humans failed. To the best of our knowledge, this presents the first assessment of general purpose LLMs on basic bioinformatics tasks in distinct areas relative to both expert and non-expert ground truths.

**Availability and Implementation:**

The complete analysis was implemented in Python; all code is available at https://github.com/SiwoResearch/lllm-bioinformatics.

## 1 Introduction

AI systems have the potential to accelerate every step of the scientific discovery process, including literature search and synthesis, hypothesis generation, research planning, execution of experiments, data collection, analysis and interpretation of results. Recently, there have been numerous studies showcasing the potential of large language models (LLMs), AI agents and co-scientists in accelerating scientific research in fields such as chemistry, material sciences and biology (Boiko *et al.* 2023, Ghareeb *et al.* 2025, Gottweis *et al.* 2025, Miret and Krishnan 2025, Swanson *et al.* 2025). Bioinformatics- computational analysis of DNA, RNA and protein sequences- is central to advancing fundamental biological knowledge and its translation to solutions in health, agriculture and manufacturing. Computational systems that perform bioinformatics analysis using instructions provided in natural language could broaden its impact and accelerate biological research. Bioinformatics analysis is particularly amenable to end-to-end automation with AI systems since it is not constrained by physical, laboratory experimentation.

Several strategies are being pursued in the development of LLMs and AI agents for performing biological research. Most of this work has focused on their capacity for retrieval of information from literature using both general purpose and biological domain specific LLMs trained on scientific literature. For example, BiomedGPT, a multimodal biomedical model, achieved strong benchmark performance using text and image inputs, yet its evaluations focused on classification and QA rather than open-ended reasoning (Luo *et al.* 2026). BioInstruct, another instruction-tuned biomedical LLM, showed improvement on structured QA but did not evaluate performance on realistic, multi-step bioinformatics tasks (Tran *et al.* 2024).

A powerful strategy to enable LLMs to perform complex tasks involves providing them access to tools and integrating them using agentic frameworks. Recent reports describe biomedical AI agents and co-scientists capable of reasoning, hypothesis generation, knowledge synthesis and execution of chemistry experiments (Boiko *et al.* 2023, Ha *et al.* 2023, Gottweis *et al.* 2025) but only a few have demonstrated capability for automated biological data analysis. For example, TxGemma was obtained by fine-tuning the generalist Gemma model on therapeutic tasks (Wang *et al.* 2025) while Google AI co-scientist leverages a multi-agent system built on Gemini 2.0 to generate hypotheses (Gottweis *et al.* 2025). TxAgent performs multi-step reasoning and retrieval of biomedical knowledge across 211 tools with a focus on therapeutic tasks (Gao *et al.* 2025), and the Virtual Lab consists of an LLM agent that acts as a principal investigator collaborating with a team of LLM scientist agents with a human in the loop (Swanson *et al.* 2025). On the other hand, Biomni, a biomedical AI agent, is capable of mining critical tools, databases and protocols that it subsequently leverages to autonomously perform a wide range of biomedical research tasks (Huang *et al.* 2025). In the case of bioinformatics related tasks, a version of Biomni (A1) with access to 150 specialized biomedical tools, 105 software packages and 59 databases was able to analyse single-cell RNA-seq and ATAQ-seq datasets based on instructions provided in natural language (Huang *et al.* 2025). An alternative approach involves training multi-modal LLMs/AI agents that integrate text and biological sequences. For example, ChatNT is a multi-modal conversational agent for biological sequence analysis tasks, addressing the gap between biological foundation models and conversational agents (de Almeida *et al.* 2025). BioReason, on the other hand, integrates a DNA foundation model with LLMs to perform multi-step reasoning on specific genomics tasks (Fallahpour *et al.* 2025).

While both generalist and biomedical domain LLMs could in principle be integrated with tools for bioinformatics analysis, understanding their ability to perform tasks out-of-the-box i.e. without further fine-tuning or specialized training is important for several reasons; (i) Generalist LLMs such as OpenAI GPT, Llama, Claude and Gemini models are widely accessible, making them the first go to tools for non-experts; (ii) As the world progresses towards artificial general intelligence (AGI), out-of-the-box bioinformatics capabilities of generalist LLMs combined with their improving capacity in a broad range of domains could enable them to discover complex solutions that integrate knowledge within and outside of biomedical sciences- from molecules to ecosystems; (iii) Identifying baseline capabilities of general purpose LLMs could also help inform their efficient and reliable integration with biological domain specific tools and agents. Though not a focus of this work, understanding out-of-the box capabilities of general purpose LLMs is critical in anticipating biosecurity risks of these models.

Here, we assess the ability of six generalist LLMs—GPT-3.5, GPT-4o, GPT-5.2, Llama-3-70B, Llama-4 and Claude Sonnet 4.6 to solve basic bioinformatics problems and compare their performance to that of humans. We contribute to the growing body of work on AI-assisted bioinformatics by evaluating LLM performance on 104 open-ended questions from Rosalind, an educational platform on practical bioinformatics problems. This study offers insights into both the strengths and limitations of generalist LLMs in performing bioinformatics tasks and advances efforts to make bioinformatics more accessible by reducing the need for specialized expertise.

### 1.1 Contributions

This work is closely related to recent efforts benchmarking the performance of LLMs in bioinformatics tasks. In particular, the BixBench benchmark dataset consisting of 53 analytical scenarios and 296 open-ended questions curated by bioinformatics experts (Mitchener *et al.* 2025), LabBench which consists of 2400 multi-choice questions on practical biology tasks with a small subset on DNA and protein sequences (Laurent *et al.* 2024), BaisBench performs assessment of LLMs on single-cell analysis tasks (Luo *et al.* 2025) and BioCoder which is a benchmark dataset for evaluating LLMs in bioinformatics-code generation (Tang *et al.* 2024). Key contributions include:

- *Assessment of LLMs vs. thousands of humans on bioinformatics analytical tasks:* We assess performance based on bioinformatics tasks undertaken by thousands of experts and non-experts.
- *Assessment of LLMs on basic, low-level bioinformatics analysis tasks*: We focus on basic‘low-level’ analytical tasks in a broad range of areas in bioinformatics that include concepts in Mendelian genetics, genetic code translation, genome assembly and phylogenetics that are foundational to being a bioinformatician.
- *Assessments of LLMs on bioinformatics tasks requiring multi-step reasoning and code writing*: This study is distinct in that it uses bioinformatics analysis problems that require multi-step reasoning, biological context interpretation, and code-like logic to arrive at an exact output.

## 2 Methods

### 2.1 Data sources

This study utilized a total of 104 questions sourced from the Bioinformatics Stronghold section of Rosalind (http://rosalind.info), a widely used online platform that teaches bioinformatics with practice problems. The questions covered key bioinformatics topics of ranging difficulty, including DNA analysis, restriction mapping and enzymes, phylogenetics, combinatorics and probability, RNA and protein translation, genome assembly and graph algorithms, sequence alignment and comparative genomics, genome analysis and computational biology, and motif finding and pattern matching. Each problem included a precise computational task and sample data, some including biological context. The answers to each question were obtained from Rosalind and used as expert ground truth. Answers to each question is a unique output that includes precise numbers and strings (e.g. DNA/RNA or protein sequence).

Question difficulty was estimated using the proportion of human users who answered each question correctly, based on Rosalind’s user statistics. These questions had been attempted by between 110 and 68 760 individuals each.

### 2.2 Large language models (LLMs)

We evaluated the following large language models (LLMs) from different model providers: OpenAI (GPT-3.5, GPT-4o, GPT-5.2), MetaAI (Llama-3-70B, Llama-4 Maverick) and Anthropic (Claude Sonnet 4.6). GPT-3.5, GPT-4o, Llama-3-70B and Llama-4 Maverick were accessed using a customized generative AI interface at the University of Michigan known as UM GPT platform. The UM GPT platform provides functionally equivalent chat interfaces to these LLMs but with additional privacy safeguards. For example, information provided to LLMs on the UM GPT platform are not shared with the servers of the model providers.

In addition to using the UM GPT platform, we also accessed GPT-4o and Claude Sonnet 4.6 programmatically via the OpenAI and Anthropic Application Programming Interfaces (APIs), respectively. For both models, we set a temperature = 1 value to control for randomness/variability in responses. We refer to the GPT-4o model accessed via API as GPT Base and Claude Sonnet 4.6 as Claude Base.

### 2.3 Evaluation of LLMs

Each question from the Rosalind platform was provided as a prompt to each LLM, keeping the prompt identical between models. Each model was tested on all 104 questions. The final output was checked for correctness. The performance of models was evaluated by computing their accuracy against solutions provided by Rosalind (expert ground truth) across all the 104 questions. Accuracy of individual answers provided by LLMs was manually assessed. In addition to overall accuracy, we computed accuracy of models based on pre-specified categories of bioinformatics questions as provided in Table S1.

To compare the relationships between the models based on patterns of correctness and incorrectness, we performed McNemar’s test and used chi-square and *P*-value to determine statistical significance Additionally, each question was categorized by its primary bioinformatics topic area to assess differences in model performance across different content areas.

All analyses were conducted using Python with the pandas, numpy, matplotlib, seaborn, and scipy libraries. Code used for the analysis is available at https://github.com/SiwoResearch/lllm-bioinformatics.

### 2.4 Assessment of LLMs relative to perceived human difficulty of questions

For each question the number of humans (Rosalind users) who have tackled the question as well as the proportion of humans who answered the question correctly was obtained from Rosalind. We categorized each question into overlapping difficulty thresholds based on human accuracy rates: questions where more than 30%, 40%, 50%, 60%, 70%, 80%, and 90% of users answered correctly. Within each benchmark group, we computed and visualized the distribution of AI correctness to assess how model performance varied with question difficulty. To visualize these performance patterns, we created grouped bar charts showing the counts of correct and incorrect AI responses across the benchmark bins. We computed accuracy of LLMs in answering the questions in each benchmark relative to the expert ground truth (Rosalind).

### 2.5 Retrieval augmented generation (RAG)

For retrieval augmented generation (RAG), we leveraged an open-source bioinformatics book (‘Bioinformatics for Beginners’) to create a vector database. Ingestion of the PDF book was performed using PyPDFLoader followed by chunking using RecursiveCharacterTextSplitter with chunk_size = 1000, chunk_overlap = 100.The document was then embedded using text-embedding-3-small and stored in a persistent FAISS vector and stored on disk to enable subsequent runs without need to re-embed. For retrieval, Top *K* = 10 chunks were retrieved based on cosine similarity (similarity_search_score). Deduplication was performed by skipping duplicate chunks from each page. Only unique remaining chunks were used for RAG. Full code for RAG is provided at https://github.com/SiwoResearch/lllm-bioinformatics. Two RAG models were created separately- GPT-4o RAG based on GPT-4o accessed via OpenAI API for embedding, search and retrieval and similarly Claude RAG based on Claude Sonnet 4.6 for embedding, search and retrieval.

## 3 Results

### 3.1 Relative performance of LLMs across all tasks

The Rosalind Bioinformatics Stronghold (http://rosalind.info) consists of 104 questions. We organized these questions into 10 categories consisting of ‘DNA analysis’ (7 questions), ‘Restriction mapping and enzymes’ (2 questions), ‘Phylogenetics’ (14 questions), ‘Combinatorics and probability’ (13 questions), ‘RNA and protein translation’ (13 questions), ‘Genome assembly and graph algorithms’ (9 questions), ‘Sequence alignment and comparative genomics’ (14 questions), ‘Genome analysis/computational biology’ (7 questions), ‘Motif finding and pattern matching’ (12 questions), ‘Genetic inheritance and population genetics’ (10 questions) and a set of 3 questions which we could not assign to a specific topic (‘Unassigned’) (Table S1).

Human performance varied widely, with correctness rates ranging from 25% to 91% for each question. For example, 25% of humans who attempted a question involving ‘Finding All Similar Motifs’ in a sequence were correct, while among the LLMs, only GPT5.2 and Llama-3-70B were correct. In contrast, 91% of humans provided a correct answer for a question on ‘Counting Disease Carriers’ based on knowledge of the Hardy-Weinberg principle. With the exception of GPT3.5, all the models provided correct answers in this question. Notably, there was a wide distribution in the number of humans who attempted each question with the most popular question being undertaken by 68 760 humans and least popular by only 110 individuals (median= 1140; mean = 5907).

Overall, GPT5.2 correctly answered 80/104 questions (77%), GPT-3.5 answered 59/104 correctly (58%), Llama-4 answered 56/104 (54%), Llama-3-70B (47%), Claude-Base model (Claude-4.6 Sonnet accessed via Anthropic API) answered 57/104 (55%) and GPT-4o via UM GPT platform answered 49/104 (47%). GPT-Base (GPT-4o accessed via OpenAI API) answered 52/104 (50%) of the questions correctly. Notably, although GPT-4o and GPT-4 Base each used the same underlying model, accessing the latter via OpenAI API allowed us to control parameters, specifically temperature which was set to zero. In contrast, accessing GPT-4o via the UM GPT platform (similar to using ChatGPT) does not allow parameters to be set and uses parameters that are not revealed to users. Models augmented by retrieval augmented generation (RAG) had lower performance than their corresponding base models (Claude RAG = 53% vs Claude Base = 77%; GPT RAG = 46% vs GPT Base = 50%). A detailed assessment of the RAG models is provided in the section on “Impact of retrieval augmented generation (RAG)”. Since LLMs can be sensitive to prompting, we also tested whether rephrasing questions led to different answers but did not observe any deviations potentially because the Rosalind questions are typically short and factual, and the answers to these questions are not open ended but precise, for example, numbers and sequences. We provide examples in the Supplementary Text.

### 3.2 Error profiles of LLMs and humans

21 questions were answered correctly by all models, and 10 were answered incorrectly by all models (Table S1). Thus, in nearly 20% of questions, all the models had the same outcome (i.e. all correct or all incorrect). 90% (94 out of 104 questions) were answered correctly by at least one of the LLMs suggesting that LLMs ensembles or mixture of experts (MoE) could potentially be a strategy for improving performance.

We further analysed the nature of tasks in which all the LLMs passed or failed. Out of 21 questions in which all LLMs passed, 4 (17%) involved ‘Phylogenetics’, another set of 4 involved ‘DNA analysis’. ‘Combinatorics and probability’ and ‘Genetic inheritance and population genetics’ each accounted for 3 (13%) questions in which all the LLMs passed. There were 2 (9%) questions under ‘Unassigned’ categories in which all the LLMs passed. For the following categories there was only 1 (4%) question in which all the LLMs passed: ‘Sequence alignment and comparative genomics’, ‘Motif finding and pattern matching’, ‘Genome assembly and graph algorithms’, ‘Genome analysis/computational biology’ and ‘RNA and protein translation’.

Out of 10 questions in which all LLMs failed, 5 (50%) involved ‘Sequence alignment and comparative genomics’. A distinct set of 2 (6%) involved ‘Genome assembly and graph algorithms’, another 2 (20%) involved ‘Motif finding and pattern matching’. and 1 (10%) involved ‘Combinatorics and probability’.

We investigated whether the performance of humans in tasks in which LLMs provide correct answers differs from that in tasks in which LLMs provide incorrect answers. Human performance tended to be higher in tasks in which LLMs provided correct answers compared to in tasks where LLMs were incorrect (Table 1).

**Table 1:** LLM and human performance.

	GPT-3.5	GPT-4o	GPT5.2	Llama-3	Llama-4	Claude-4.6	All LLMs
Average proportion of correct humans in AI correct	0.64	0.63	0.64	0.62	0.64	0.65	0.67
Average proportion of correct humans in AI incorrect	0.59	0.60	0.53	0.61	0.58	0.57	0.55

### 3.3 Relationships between models based on performance

To assess relationships between models in terms of their correctness and incorrectness, we determined cases where one model was correct and the other was incorrect and vice versa. McNemar’s test was used to determine whether model pairs were significantly different from each other on this basis (Fig. 1). Interestingly, GPT-5.2 vs. GPT-4o, and GPT-5.2 vs. Llama-3 had significantly different error patterns (McNemar’s chi-square statistic = 22; *P* < .001; Fig. 1). Specifically, there were 36 questions in which GPT-5.2 was correct while GPT-4o was wrong and only 5 questions in which GPT-4o was correct while GPT-5.2 was wrong. Llama-3 was correct in 5 questions in which GPT-5.2 was incorrect while GPT-5.2 was correct in in 36 questions in which Llama-3 was incorrect. Overall, GPT-5.2 was the most significantly different from all the other models based on its ability to provide correct answers where other models were incorrect (McNemar’s test *P* = .001; Fig. 1). None of the other models was significantly different from each other (McNemar’s test *P* > .05).

**Figure 1:**
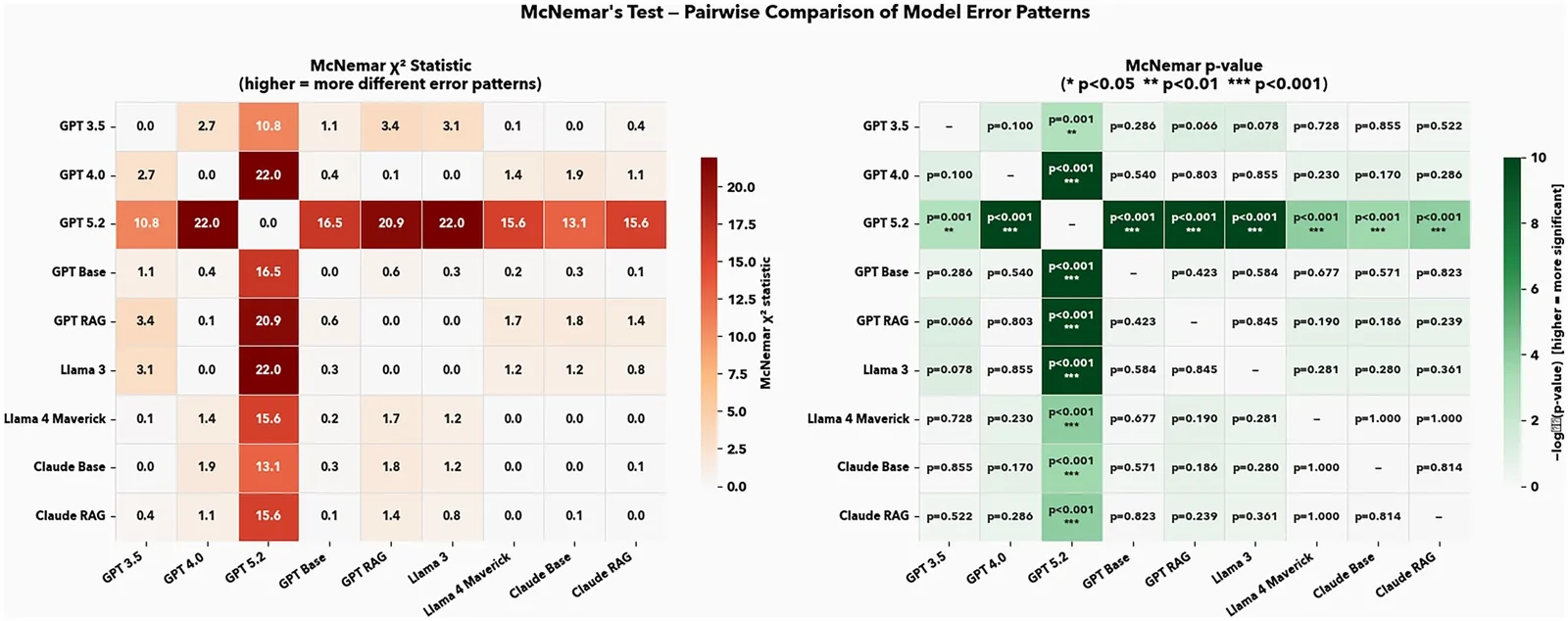
Comparisons of the LLM models based on patterns of correctness and incorrectness.

Next, we asked whether certain questions were only correctly answered by one model and found that only 10 met this criterion. Out of these, there were 6 (60%) questions in which only GPT-5.2 provided correct answers compared to 1 for Claude Base, 1 for GPT-4o, 1 for GPT Base and 1 for Llama-4. For the questions where GPT-5.2 was the only model with correct answers, 2 involved ‘Genome analysis/computational biology’, and 1 each involved ‘Genetic inheritance and population genetics’, ‘Genome assembly and graph algorithms’, ‘RNA and protein translation’, and ‘Sequence alignment and comparative genomics’.

### 3.4 Performance across biological categories of questions

Performance of the LLM models varied widely across biological categories. Top-performing categories across the models were ‘DNA analysis’ (GPT-3.5, GPT-Base, Claude-RAG: 100%; Llama 3, Llama-4: 86%, GPT-4o: 57.1%), and ‘Restriction mapping and enzymes’ (GPT-3.5, GPT-RAG, Claude-Base, Claude-RAG: 100%; Fig. 2). Notably, there were very few questions in these categories which biases the results. Lower-performance categories included ‘Sequence Alignment’ (GPT-5.2 accuracy: 57%; other models <50%; Fig. 2) and ‘Motif finding and pattern matching’ (GPT-5.2 and Llama-4: 58%; all other models <50%; Fig. 2).

**Figure 2:**
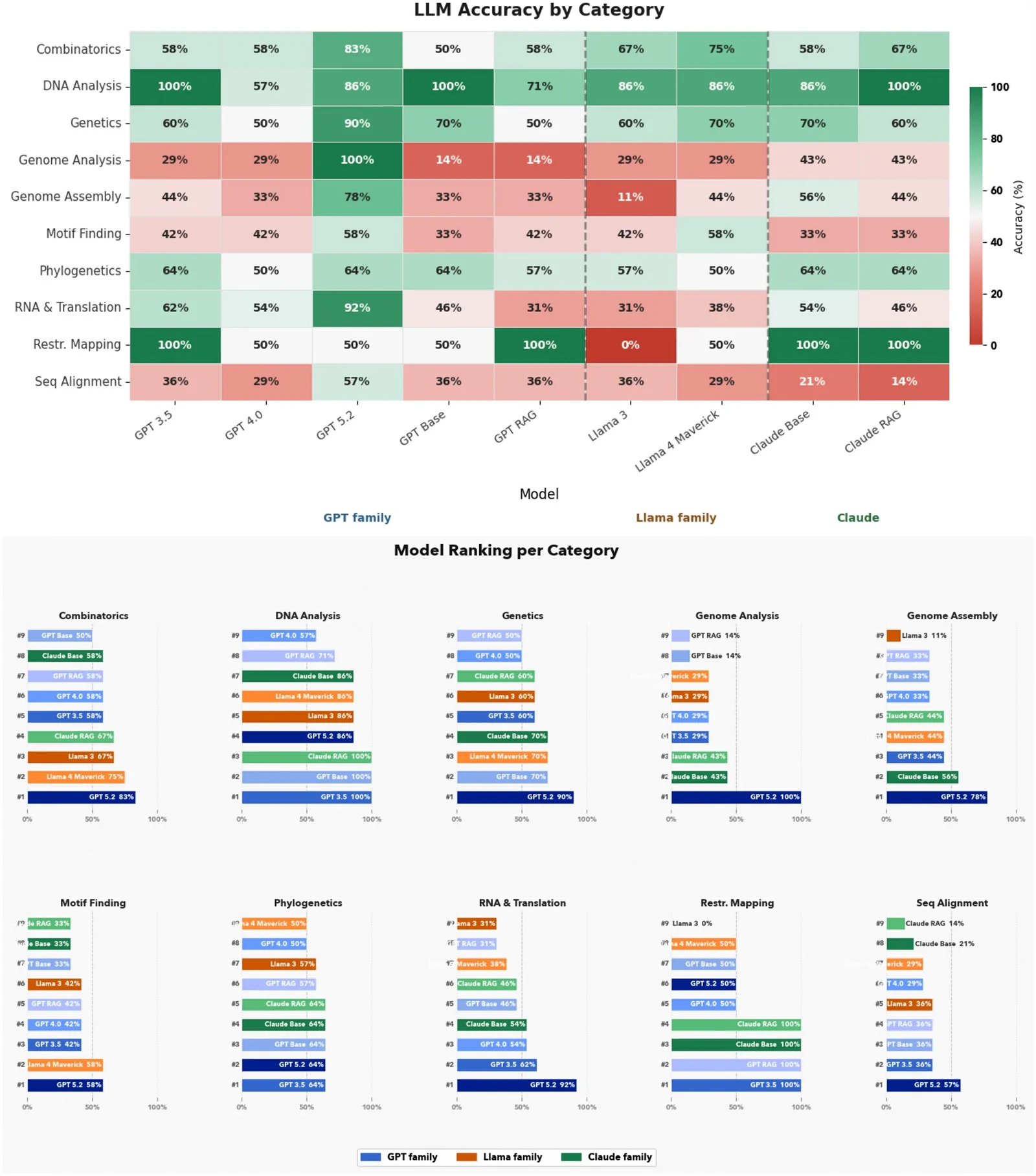
Performance of the LLM models across different categories of bioinformatics tasks.

GPT-5.2 was the top model across multiple categories including ‘Combinatorics’ (accuracy = 83%), ‘Genetics’ (accuracy = 90%), ‘Genome Analysis’ (accuracy = 100%), ‘Genome Assembly’ (78%), ‘Motif finding’ (58%), ‘RNA and Protein Translation’ (92%) and ‘Sequence Alignment’ (57%). No model was consistently the worst performing (Fig. 2).

### 3.5 Performance of LLMs at different benchmarks of human difficulty

We next investigated the performance of LLMs in tasks in which humans had high vs. low performance. The top 20 questions in which humans had the highest performance (average of 82% of humans correct) involved ‘Genetic inheritance and population genetics’ (4 questions), ‘DNA analysis’ (2 questions), ‘Combinatorics and probability’ (3 questions), ‘Motif finding and pattern matching’ (1 question), ‘RNA and protein analysis’ (1 question), ‘Restriction mapping and enzymes’ (1 question), ‘Phylogenetics’ (1 question), ‘Unassigned’ (1 question). Among these top 20 tasks in humans, all the LLMs were correct in 5 cases (25%) and incorrect in 4 cases (20%). GPT-5.2 was correct in all the top 20 cases (100%), GPT-3.5 was correct in 12 cases (60%), GPT-4o in 11 cases (55%), and Llama-3-70B in 8 cases (40%).

In contrast, the top 20 questions in which humans had the lowest performance (average 41% humans provided correct answers) involved: ‘Motif finding and pattern matching’ (3 questions), ‘Sequence alignment and comparative genomics’ (8 questions), ‘Phylogenetics’ (4 questions), ‘RNA and protein translation’ (2 questions), ‘Genome analysis/Computational biology’ (1 question), and ‘Combinatorics and probability’ (2 questions). Among these tasks where humans had low performance, all the LLMs were correct in 0 cases and incorrect in 3 cases. GPT-5.2 was correct in 13 cases, GPT-4o was correct in 6 cases, GPT-3.5 was correct in 7 cases, GPT Base in 8 cases, and Claude Base, Llama-3 and Llama-4 were each correct in 7 cases. Thus, LLMs had high performance in questions in which human performed highly and vice versa.

We hypothesized that the number of humans who undertook a given question is another dimension that reflects the potential difficulty of the questions to humans. On average, 64% of humans provided correct answers among the top 20 most attempted questions compared to 52% correct answers in the 20 least attempted questions. This implies that human tendency to undertake the questions likely reflects perceived level of difficulty. Therefore, we next assessed the performance of LLMs based on this perceived level of difficulty. Among the top 20 most attempted questions (average number of humans = 24 456), 8 were correctly answered by all the LLMs and one was incorrectly answered by all the LLMs. In contrast, among the least 20 answered questions (average number of humans = 225), only one was correctly answered by all the LLMs and 5 were incorrectly answered by all LLMs. This further supports the hypothesis that LLMs performance is lower on questions that are either less popular or are perceived as difficult by humans.

To better understand the relationship between LLM and human performance, we established different benchmarks of human performance based on the proportion of humans who provided a correct response to each question. GPT-5.2 is the only model that had more correct than incorrect answers at all benchmarks (Fig. 3). Claude Base had more correct than incorrect answers at most benchmarks except at 30–39 and 50–59. GPT-3.5 had more correct than incorrect responses at benchmarks from 30–79, while GPT-4o and Llama-3-70B provided more incorrect answers at most benchmarks except for the 70–79 benchmark (Fig. 3).

**Figure 3:**
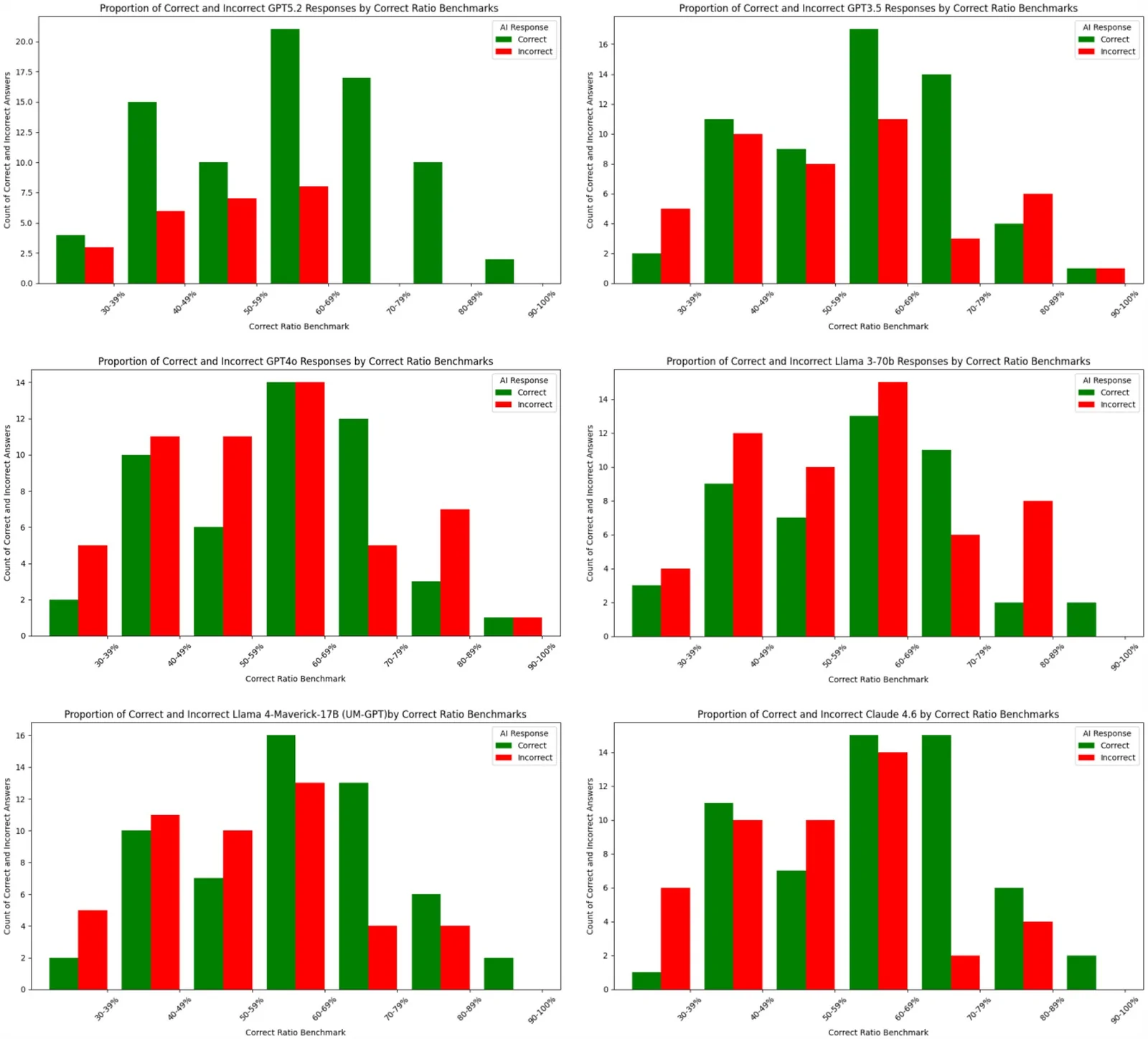
Performance of LLMs at different benchmarks of human performance.

### 3.6 Performance across model generations

LLM model developers continuously update their models to newer versions, generally with expected model improvements. However, there is no guarantee that newer models with overall performance improvements will show improvements in all tasks. Among the models evaluated in this study, GPT-5.2 is the successor of GPT-4 while GPT-3.5 was succeeded by GPT-4. In our assessment, GPT-3.5 had slightly lower performance than GPT-4o but GPT-5.2 had significantly higher performance than both GPT-4o and GPT-3.5. For Llama models, Llama-4 was a slight improvement from Llama-3 (Fig. 4 scatter plot).

**Figure 4:**
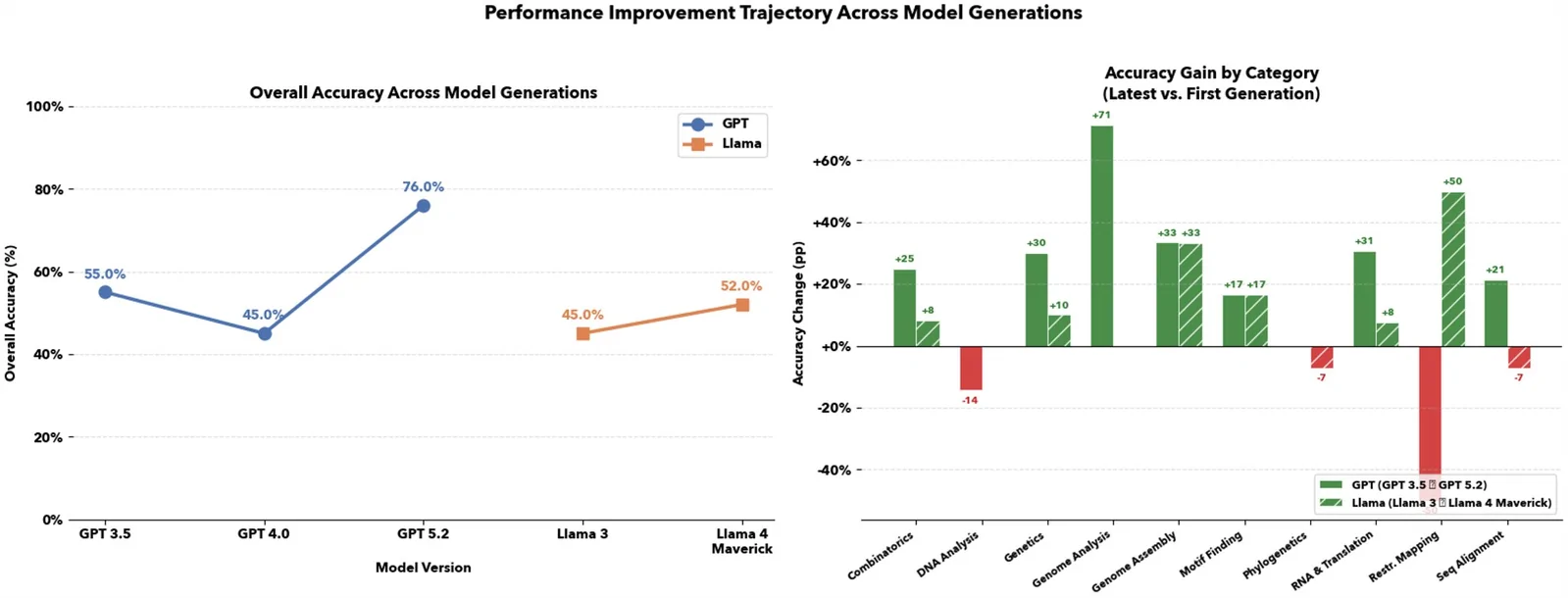
Performance across model generations. Green bar plots indicate increase in performance of latest generation model vs. first-generation models based on performance within different categories of the questions. Red bar plots indicate reduction in model performance. No change in performance appears as a ‘missing’ bar in the bar plot.

To investigate whether these improvements are sensitive to specific categories of questions, we evaluated performance of first-generation models to latest generation models. While in overall GPT-5.2 had higher performance than its first-generation counterpart (GPT-3.5), it had a 14% drop in performance among questions involving ‘DNA analysis’ and a 50% drop in questions involving ‘Restriction mapping’ (Fig. 4 bar plots). While Llama-4 was an overall improvement over Llama-3, among questions involving ‘Phylogenetics’ we observed a 7% drop and a similar drop in performance among questions on ‘Sequence alignment’.

Latest generation models had unequal improvements across question categories. For example, while for GPT-5.2 the largest improvement in performance was in ‘Genome analysis’ (71% improvement over GPT-3.5), for Llama-4, the largest improvement was on ‘Restriction mapping’. For a number of categories of questions, there was no change in performance in either model generation. For example, Llama series did not show any change in performance for the questions on “DNA analysis” while GPT series had no difference in performance in ‘Phylogenetics’ questions.

### 3.7 Impact of retrieval augmented generation (RAG)

Since many bioinformatics tasks require background biological knowledge (e.g. genetic code table to perform RNA to protein translation), we investigated whether providing LLMs with biological knowledge using a bioinformatics textbook (‘Bioinformatics for beginners’) (Choudhuri and Kotewicz 2014) could enhance performance. As shown in Fig. 5, our RAG models based on GPT-4o (GPT4 RAG) and Claude 4.6 (Claude RAG) did not perform better than their corresponding base models when considering all questions. We reasoned that RAG may provide better performance depending on the context of a question and therefore evaluated performance of the RAG models on questions from the different biological categories. For GPT-4o, RAG provided an improvement over the base model in ‘Combinatorics and probability’ (+8% improvement in accuracy, Fig. 6), ‘Motif finding’ (+9%), ‘Restriction mapping’ (+50%). However, GPT RAG had reduced performance in ‘DNA analysis’ (−29%), ‘Genetics’ (−20%), ‘Phylogenetics’ (−7%) and ‘RNA and Translation’ (−15%). Performance in other categories did not change when using RAG. For Claude RAG, improvements occurred in ‘Combinatorics and probability’ (+8%) and ‘DNA analysis’ (+14%). Claude RAG performance reduced in ‘Genetics’ (−10%), ‘Genome assembly’ (−11%), ‘RNA and Translation’ (−8%) and ‘Sequence alignment’ (−7%). Performance did not change for ‘Genome analysis’, ‘Motif finding’, ‘Phylogenetics’ and ‘Restriction mapping’ for Claude RAG.

**Figure 5:**
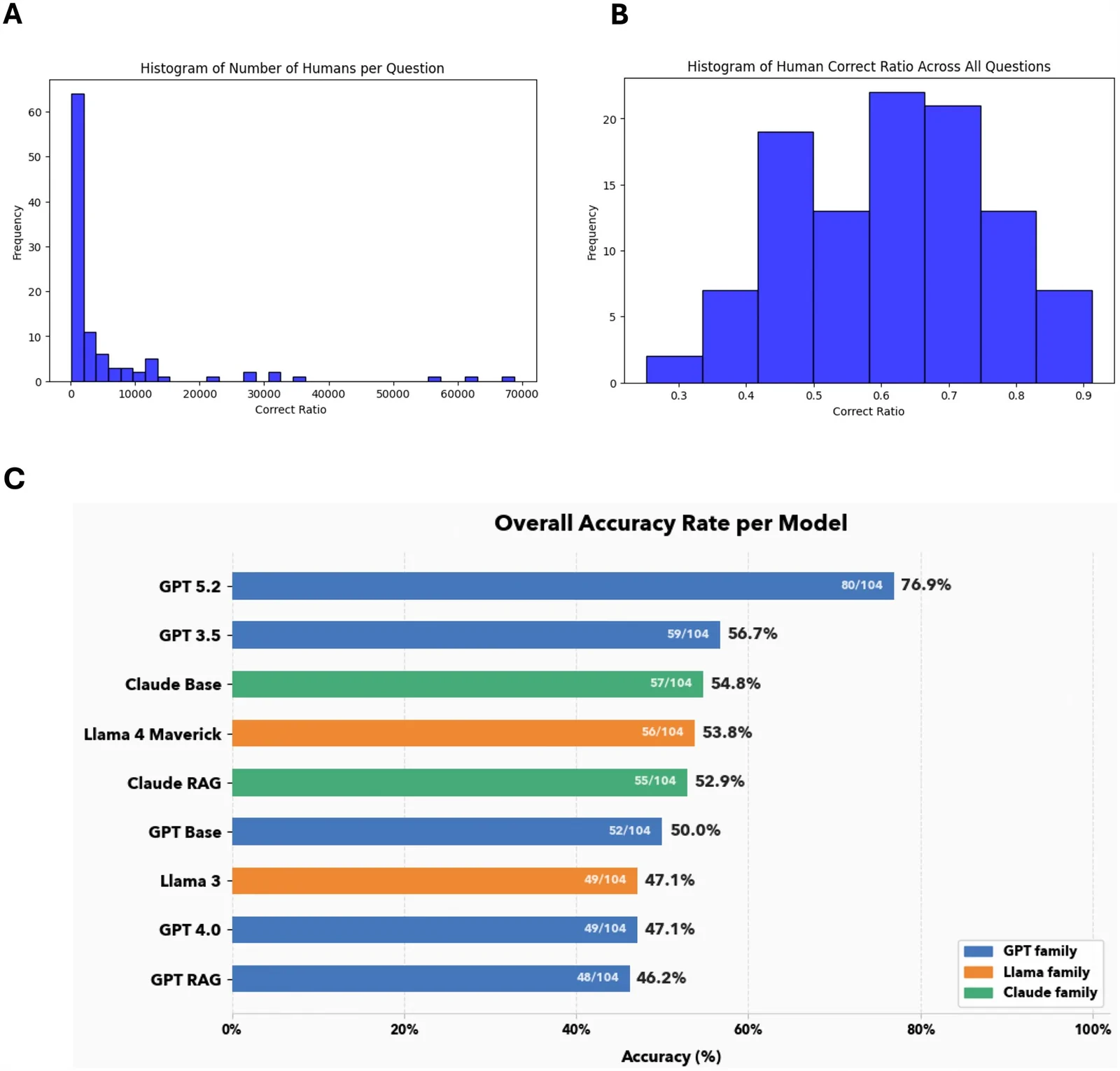
Summary of human and LLM performance on the Rosalind Bioinformatics Stronghold dataset. (A) Number of humans who provided answers per question. (B) Distribution of the average proportion of humans who provided a correct answer to each question (Correct Ratio). (C) Performance of LLMs (accuracy) as assessed based on expert (Rosalind) ground truth. GPT-Base is GPT-4o accessed via OpenAI API, Claude-Base is Claude Sonnet 4.6 accessed via Anthropic API.

**Figure 6:**
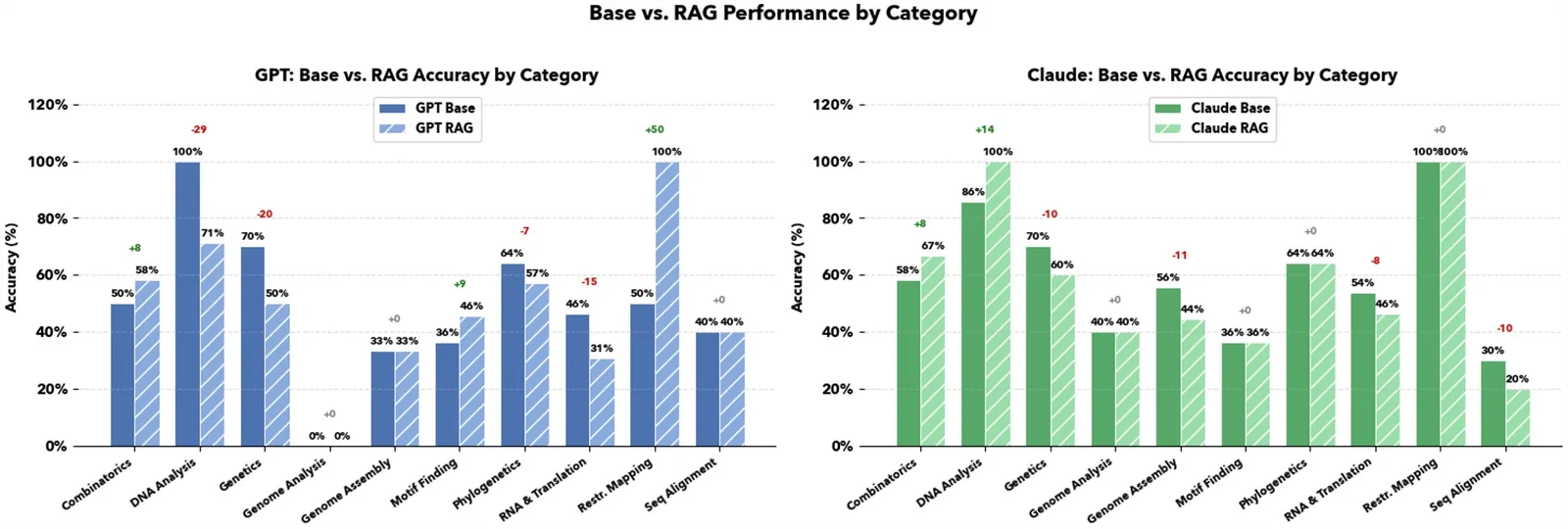
Performance of RAG models across biological categories of questions.

### 3.8 Analysis of reasoning traces and errors

To gain a deeper understanding of potential sources of error for the LLMs on specific tasks, we assessed the reasoning traces/explanations and code provided by the LLMs. LLMs have been shown to not follow their exact chain of thought leading to mismatch between explanations provided and answers provided by LLMs.

For example, examining the reasoning traces/chain of thought of the LLMs on a question on ‘RNA to protein translation’ given an mRNA sequence as input, GPT-4o reasoned correctly that translation is initiated from a start codon (AUG), proceeds in triplets and ends at a stop codon, with each amino acid encoded by a codons based on a standard genetic code that maps each amino acid to its corresponding codon (Supplementary Text). Despite this correct reasoning including identifying the correct amino acids encoded by the provided sequence in the right order, GPT-4o’s final answer contained a small typo leading to an incorrect protein sequence- ‘MA**A**MAPRTIESTN**I**RG’ instead of ‘MAMAPRTEINSTRING’. GPT Base had a partly correct chain of thought but appears to have skipped one nucleotide near the end of the sequence resulting in a reading frame change and truncated peptide sequence - “MAAPRTEINSTRI”. Interestingly, GPT RAG was able to perform correct reasoning and provide the correct answer. Claude models (Claude 4.6 Sonnet and Claude RAG) both had highly similar reasoning and produced the correct response (Supplementary Text). We provide additional examples in the Supplementary Text that includes cases involving writing code in which one or more LLMs had the right logic but only provided a partial solution as well as cases where the answers were correct or incorrect. To enhance transparency, we provide a JSON file in the Supplementary Material with all the prompts, reasoning traces and responses generated by the models.

## 4 Discussion and conclusion

Understanding LLM performance on basic bioinformatics tasks has not been extensively evaluated yet is critical to performance of complex bioinformatics AI agents which perform specialized tasks such as single cell RNA-seq analysis. For AI agents to be effective, they need to be able to understand basic tasks which are typically not performed by specialized bioinformatics tools. Furthermore, given the natural language capabilities of general purpose LLMs, out-of-the-box capabilities in these models could enhance their integration with specialized biomedical AI agents and bioinformatics tools while benefiting from rapid progress in non-biomedical LLMs. Despite the explosion of biomedical domain LLMs and AI agents, most of these systems are developed and evaluated for specialized biomedical tasks, do not take natural language inputs, and are intended for domain experts, limiting their broad utility.

This study explored the capabilities of the large language models GPT-3.5, GPT-4o, GPT-5.2, Llama 3-70B, Llama-4 and Claude Sonnet 4.6 in performing bioinformatics tasks relative to humans with a wide range of expertise levels. While each model correctly answered 46 to 77% of the 104 open-ended questions sourced from the Rosalind Bioinformatics Stronghold, the models also failed in some questions where most humans obtained the correct answer. It is notable that the performance of LLMs was very dependent on the performance of humans on specific tasks- tasks in which humans had high performance were well done by the LLMs compared to those in which humans had low performance. A dependency on the number of humans who undertook a given question was also evident with LLMs showing high performance on tasks that were undertaken by many humans compared to performance in tasks that fewer humans undertook. Thus, it appears that the number of humans who undertook a given question reflects the perceived difficulty of the question.

LLMs demonstrated strong performance in areas such as DNA analysis and restriction mapping—domains that often involve more straightforward computations or well-defined rules. Conversely, they struggled in categories that require higher-order reasoning, algorithm selection, or pattern recognition, such as genome assembly, phylogenetics, motif finding, and sequence alignment. Despite these limitations, the models exhibited notable proficiency in generating answers that required a combination of biological reasoning, statistical analysis, and computational logic. Their ability to solve complex problems without prior specialization in bioinformatics underscores the potential for LLMs to assist in this domain, particularly for educational or exploratory use cases. However, the models’ occasional production of incomplete or incorrect answers highlights current gaps in training and the need for improved domain adaptation.

The analysis of first and latest generations of LLM models revealed that while latest generation models tend to have better overall performance, their improvements are not equally distributed across questions from distinct biological categories. For example, while GPT-5.2 showed high improvements in ‘Genome analysis’ related questions relative to GPT-3.5, its performance on questions on DNA analysis was lower than that of GPT-3.5. Thus, it is important for the assessment of models on bioinformatics tasks to consider how they perform on tasks from distinct biological categories.

This study is limited by the fact that the bioinformatics tasks presented are not comprehensive enough to determine whether one LLM is better than the other. While GPT-5.2 had the highest performance, this may not be generalizable to performance on other bioinformatics tasks. It is important to note that the tasks in Rosalind require multi-step reasoning using different aspects of biological knowledge. Thus, although the number of questions tested is relatively small, the extent of bioinformatics knowledge covered by the questions is much larger. Future work will focus on extending the evaluations to include the latest versions of LLMs and more bioinformatics tasks, assessing reasoning traces/chain-of-thought, enhancing LLM performance using mixture of experts, and investigating biosecurity risks.

## Supplementary Material

btag749_Supplementary_Data

## Data Availability

All code used for data preprocessing, analysis, and visualization was written in Google Colab. The complete analysis was implemented in Python. https://github.com/SiwoResearch/lllm-bioinformatics.
