# Supplementary material for: Assessment of LLMs on basic bioinformatics tasks requiring multi-step reasoning and code writing
Source: Bioinformatics. 2026 Oct 30;42(10):btag749. doi: 10.1093/bioinformatics/btag749 (PMC13722031; doi:10.1093/bioinformatics/btag749)
Supplement: Supplementary file 1 [file btag749_supplementary_data.zip › Supplementary_text_example_reasoning.docx]

**Rephrasing questions**

Each of the questions below was presented to Claude 4.6 in an original form (exactly matching the question from Rosalind) and in rephrased forms. The answers provided by Claude 4.6 when using the original form of the question was then compared to answers from the Rephrased forms.

**Claude 4.6**

**Original question 1**: "Given: A DNA string s of length at most 1000 nt. Return: Four integers (separated by spaces) counting the respective number of times that the symbols 'A', 'C', 'G', and 'T' occur in s. Sample Dataset AGCTTTTCATTCTGACTGCAACGGGCAATATGTCTCTGTGTGGATTAAAAAAAGAGTGTCTGATAGCAGC"

Output: 20 12 17 21

**Rephrased question 1:** Given: A DNA string s of length at most 1000 nt. Return: Four integers (separated by spaces) counting the respective number of times that the symbols 'A', 'C', 'G', and 'T' occur in s. Sample Dataset AGCTTTTCATTCTGACTGCAACGGGCAATATGTCTCTGTGTGGATTAAAAAAAGAGTGTCTGATAGCAGC"

Output: 20 12 17 21

**Rephrased question 1**: Given: A DNA string s of length at most 1000 nt. Please provide 4 numbers that reflect how frequently 'A', 'C', 'G', and 'T' occur in s. Sample Dataset AGCTTTTCATTCTGACTGCAACGGGCAATATGTCTCTGTGTGGATTAAAAAAAGAGTGTCTGATAGCAGC"

20 12 17 21

Detailed Breakdown:

A: 20 (28.57%)

C: 12 (17.14%)

G: 17 (24.29%)

T: 21 (30.00%)

######################################################################

**Original question 2:** Given: A DNA string t having length at most 1000 nt. Return: The transcribed RNA string of t. Sample Dataset GATGGAACTTGACTACGTAAATT"

\# Output: GAUGGAACUUGACUACGUAAAUU

**Rephrased question 2:** Given: A DNA string t having length at most 1000 nt. Return: What is the result of transcribing this sequence? Sample Dataset GATGGAACTTGACTACGTAAATT"

\# Output: GAUGGAACUUGACUACGUAAAUU

**Rephrased question 2:** A DNA string t having 1000 base pairs. What would be the resulting RNA sequence from transcription of t. Sample Dataset GATGGAACTTGACTACGTAAATT"

\# Output: GAUGGAACUUGACUACGUAAAUU

**############################################################################**

**Example prompts and reasoning traces**

**RNA to Protein Translation reasoning**

**GPT-4o example**

"Given: An RNA string s corresponding to a strand of mRNA (of length at most 10 kbp). Return: The protein string encoded by s.

Sample Dataset AUGGCCAUGGCGCCCAGAACUGAGAUCAAUAGUACCCGUAUUAACGGGUGA"

Answer: To determine the protein string encoded by the RNA sequence, you need to translate it into a sequence of amino acids. This involves using the genetic code, which specifies which codons (triplets of nucleotides) correspond to which amino acids.

Here is a table of the standard genetic code:

UUU -> F (Phenylalanine)    CUU -> L (Leucine)       AUU -> I (Isoleucine)       GUU -> V (Valine)

UUC -> F (Phenylalanine)    CUC -> L (Leucine)       AUC -> I (Isoleucine)       GUC -> V (Valine)

UUA -> L (Leucine)          CUA -> L (Leucine)       AUA -> I (Isoleucine)       GUA -> V (Valine)

UUG -> L (Leucine)          CUG -> L (Leucine)       AUG -> M (Methionine, start) GUG -> V (Valine)

UCU -> S (Serine)           CCU -> P (Proline)       ACU -> T (Threonine)        GCU -> A (Alanine)

UCC -> S (Serine)           CCC -> P (Proline)       ACC -> T (Threonine)        GCC -> A (Alanine)

UCA -> S (Serine)           CCA -> P (Proline)       ACA -> T (Threonine)        GCA -> A (Alanine)

UCG -> S (Serine)           CCG -> P (Proline)       ACG -> T (Threonine)        GCG -> A (Alanine)

UAU -> Y (Tyrosine)         CAU -> H (Histidine)     AAU -> N (Asparagine)       GAU -> D (Aspartic acid)

UAC -> Y (Tyrosine)         CAC -> H (Histidine)     AAC -> N (Asparagine)       GAC -> D (Aspartic acid)

UAA -> Stop                 CAA -> Q (Glutamine)     AAA -> K (Lysine)           GAA -> E (Glutamic acid)

UAG -> Stop                 CAG -> Q (Glutamine)     AAG -> K (Lysine)           GAG -> E (Glutamic acid)

UGU -> C (Cysteine)         CGU -> R (Arginine)      AGU -> S (Serine)           GGU -> G (Glycine)

UGC -> C (Cysteine)         CGC -> R (Arginine)      AGC -> S (Serine)           GGC -> G (Glycine)

UGA -> Stop                 CGA -> R (Arginine)      AGA -> R (Arginine)         GGA -> G (Glycine)

UGG -> W (Tryptophan)       CGG -> R (Arginine)      AGG -> R (Arginine)         GGG -> G (Glycine)

Given the RNA sequence:

AUGGCCAUGGCGCCCAGAACUGAGAUCAAUAGUACCCGUAUUAACGGGUGA

Let's translate it into the protein string, codon by codon:

- AUG -> M (Methionine, start)
- GCC -> A (Alanine)
- AUG -> M (Methionine)
- GCG -> A (Alanine)
- CCC -> P (Proline)
- AGA -> R (Arginine)
- ACU -> T (Threonine)
- GAG -> E (Glutamic acid)
- AUC -> I (Isoleucine)
- AAU -> N (Asparagine)
- AGU -> S (Serine)
- ACC -> T (Threonine)
- CGU -> R (Arginine)
- AUU -> I (Isoleucine)
- AAC -> N (Asparagine)
- GGG -> G (Glycine)
- UGA -> Stop (Translation stops here)

So, the resulting protein string encoded by the RNA sequence is:

MAAMAPRTIESTNIRG

**Analysis: incorrect, adds an extra A at beginning and switches N and I**

##############################################################################

**GPT Base**

"Given: An RNA string s corresponding to a strand of mRNA (of length at most 10 kbp). Return: The protein string encoded by s.

Sample Dataset AUGGCCAUGGCGCCCAGAACUGAGAUCAAUAGUACCCGUAUUAACGGGUGA"

To translate an mRNA sequence into a protein string, we need to follow the process of translation, which involves converting the nucleotide sequence into a sequence of amino acids. This is done using the genetic code, where each set of three nucleotides (codon) corresponds to a specific amino acid or a stop signal.

Here's how you can translate the given mRNA sequence

1. **Identify the Start Codon**: Translation begins at the start codon, which is typically "AUG". This codon also codes for the amino acid Methionine (M).
2. **Read Codons Sequentially**: After the start codon, read the mRNA sequence in sets of three nucleotides (codons) and translate each codon into its corresponding amino acid using the standard genetic code.
3. **Stop at Stop Codons**: Translation continues until a stop codon is encountered. The stop codons are "UAA", "UAG", and "UGA". These do not code for any amino acids and signal the termination of protein synthesis.

Let's apply this to the given mRNA sequence:

- Start with the sequence: AUGGCCAUGGCGCCCAGAACUGAGAUCAAUAGUACCCGUAUUAACGGGUGA

Translate each codon:

- AUG -> M (Methionine)

- GCC -> A (Alanine)

- AUG -> M (Methionine)

- GCG -> A (Alanine)

- CCC -> P (Proline)

- AGA -> R (Arginine)

- ACU -> T (Threonine)

- GAG -> E (Glutamic Acid)

- AUC -> I (Isoleucine)

- AAU -> N (Asparagine)

- AGU -> S (Serine)

- ACC -> T (Threonine)

- CGU -> R (Arginine)

- AUU -> I (Isoleucine)

- ACG -> T (Threonine)

- GGU -> G (Glycine)

- GA -> Stop (UGA is a stop codon)

The translation stops at the first stop codon encountered. Therefore, the protein string encoded by the given mRNA sequence is:

|  | "MAAPRTEINSTRI" |
| --- | --- |

**Incorrect answer-** skipped 1 nucleotide resulting in a change in reading frame and truncation of peptide sequence.

**GPT RAG**

"Given: An RNA string s corresponding to a strand of mRNA (of length at most 10 kbp). Return: The protein string encoded by s.

Sample Dataset AUGGCCAUGGCGCCCAGAACUGAGAUCAAUAGUACCCGUAUUAACGGGUGA"

|  | "To solve this problem, we need to translate the given RNA string into a protein string. This involves converting each codon (a sequence of three nucleotides) in the RNA string into its corresponding amino acid using the standard genetic code.  The standard genetic code is as follows:  - AUG codes for Methionine (M) and is also the start codon.  - UAA, UAG, and UGA are stop codons and do not code for any amino acid.  Here is the translation of the given RNA string:  1. AUG -> M (Methionine)  2. GCC -> A (Alanine)  3. AUG -> M (Methionine)  4. GCG -> A (Alanine)  5. CCC -> P (Proline)  6. AGA -> R (Arginine)  7. ACU -> T (Threonine)  8. GAG -> E (Glutamic Acid)  9. AUC -> I (Isoleucine)  10. AAU -> N (Asparagine)  11. AGU -> S (Serine)  12. ACC -> T (Threonine)  13. CGU -> R (Arginine)  14. AUU -> I (Isoleucine)  15. AAC -> N (Asparagine)  16. GGG -> G (Glycine)  17. UGA -> Stop  The translation stops at the first stop codon encountered, which is UGA in this case.  Therefore, the protein string encoded by the given RNA string is: “MAMAPRTEINSTRING" |
| --- | --- |

**Correct Answer**

**##############################################################################**

**Claude 4.6**

"Given: An RNA string s corresponding to a strand of mRNA (of length at most 10 kbp). Return: The protein string encoded by s.

Sample Dataset AUGGCCAUGGCGCCCAGAACUGAGAUCAAUAGUACCCGUAUUAACGGGUGA"

"I need to translate the given RNA string into a protein sequence using the genetic code.

Let me break this down step by step:

1. First, I need to identify the reading frame. Since this is an mRNA string, I should look for start codons (AUG) and translate until I hit a stop codon.

2. The genetic code translates codons (3 nucleotides) into amino acids:

- AUG = Methionine (M) - also the start codon

- Stop codons are UAA, UAG, UGA

3. Let me examine the given RNA string:

AUGGCCAUGGCGCCCAGAACUGAGAUCAAUAGUACCCGUAUUAACGGGUGA

4. I'll start from the first AUG and translate in triplets:

- AUG = M (Methionine)

- GCC = A (Alanine)

AUG = M (Methionine)

- GCG = A (Alanine)

- CCC = P (Proline)

- AGA = R (Arginine)

- ACU = T (Threonine)

- GAG = E (Glutamic acid)

- AUC = I (Isoleucine)

- AAU = N (Asparagine)

- AGU = S (Serine)

- ACC = T (Threonine)

- CGU = R (Arginine)

- AUU = I (Isoleucine)

- AAC = N (Asparagine)

- GGG = G (Glycine)

- UGA = Stop codon

5. The translation stops at the first stop codon (UGA).

So the protein sequence is: MAMAPRTEINSTRINGS

Wait, let me double-check this translation:

AUG-GCC-AUG-GCG-CCC-AGA-ACU-GAG-AUC-AAU-AGU-ACC-CGU-AUU-AAC-GGG-UGA

- AUG = M

- GCC = A

- AUG = M

- GCG = A

- CCC = P

- AGA = R

- ACU = T

- GAG = E

- AUC = I

- AAU = N

- AGU = S

- ACC = T

- CGU = R

- AUU = I

- AAC = N

- GGG = G

- UGA = Stop"

"MAMAPRTEINSTRING"

**Correct Answer**

**Claude RAG**

"Given: An RNA string s corresponding to a strand of mRNA (of length at most 10 kbp). Return: The protein string encoded by s.

Sample Dataset AUGGCCAUGGCGCCCAGAACUGAGAUCAAUAGUACCCGUAUUAACGGGUGA"

|  | "Looking at this problem, I need to translate an RNA string into a protein string using the genetic code.  Let me break down the approach:  1. Find the start codon (AUG)  2. Translate codons (groups of 3 nucleotides) into amino acids  3. Stop at the first stop codon (UAA, UAG, UGA)  Given RNA string: AUGGCCAUGGCGCCCAGAACUGAGAUCAAUAGUACCCGUAUUAACGGGUGA  First, I'll locate the start codon AUG:  - Position 0: AUG ✓ (start codon found)  Now I'll translate from this position in groups of 3:  Position 0-2: AUG → Methionine (M)  Position 3-5: GCC → Alanine (A)  Position 6-8: AUG → Methionine (M)  Position 9-11: GCG → Alanine (A)  Position 12-14: CCC → Proline (P)  Position 15-17: AGA → Arginine (R)  Position 18-20: ACU → Threonine (T)  Position 21-23: GAG → Glutamic acid (E)  Position 24-26: AUC → Isoleucine (I)  Position 27-29: AAU → Asparagine (N)  Position 30-32: AGU → Serine (S)  Position 33-35: ACC → Threonine (T)  Position 36-38: CGU → Arginine (R)  Position 39-41: AUU → Isoleucine (I)  Position 42-44: AAC → Asparagine (N)  Position 45-47: GGG → Glycine (G)  Position 48-50: UGA → Stop codon (translation ends)  The translation stops at the first stop codon UGA." |
| --- | --- |

"MAMAPRTEINSTRINGS"

**Answer Correct**

**Example where LLM answer was incorrect due to provide partial answer**

**Identifying Reversing Substitutions**

**GPT-4o**

**Given:** A [rooted binary tree](https://rosalind.info/glossary/rooted-binary-tree/) T with labeled nodes in [Newick format](https://rosalind.info/glossary/newick-format/), followed by a collection of at most 100 [DNA strings](https://rosalind.info/glossary/dna-string/) in [FASTA format](https://rosalind.info/glossary/fasta-format/) whose labels correspond to the labels of T. We will assume that the DNA strings have the same length, which does not exceed 400 [bp](https://rosalind.info/glossary/base-pair/)).

**Return:** A list of all reversing substitutions in T (in any order), with each substitution encoded by the following three items:

- the name of the species in which the symbol is first changed, followed by the name of the species in which it changes back to its original state
- the [position](https://rosalind.info/glossary/position/) in the string at which the reversing substitution occurs; and
- the reversing substitution in the form original_symbol->substituted_symbol->reverted_symbol.

Sample Dataset

(((ostrich,cat)rat,mouse)dog,elephant)robot;

>robot

AATTG

>dog

GGGCA

>mouse

AAGAC

>rat

GTTGT

>cat

GAGGC

>ostrich

GTGTC

>elephant

AATTC

To solve the problem of identifying reversing substitutions in the given rooted tree \( T \) with associated genetic strings, you need to follow these steps:

1. **Parse the Tree**: Convert the Newick format of the tree into a structured format (like a dictionary or a custom tree class) to easily traverse parent-child relationships.

2. **Parse FASTA Sequences**: Read the FASTA format to associate each node in the tree with its corresponding DNA string.

3. **Identify Paths and Reversing Substitutions**: Iterate through each position in the DNA strings:

   - Traverse each parent-child relationship path from the root to all leaves. Identify when a substitution and a reverse substitution occur.

4. **Check for Reversing Substitutions**:

   - For a parent-child pair \((s, t)\):

     - Check each position \( i \) in the strings.

     - Detect positions where a substitution is followed by a reversion:

       - Substitution occurs when \( s[i] \neq t[i] \).

       - Reversion occurs when later on the path, another child or leaf \((v, w)\) has the pattern: \( s[i] = w[i] \neq v[i] = t[i] \).

5. **Output**: For each reversing substitution, output the first changed species and reverted species names, the position, and the substitution pattern.

Let's go through an example implementation in Python:

```python

from Bio import Phylo

from io import StringIO

from Bio import SeqIO

newick_tree = "(((ostrich,cat)rat,mouse)dog,elephant)robot;"

fasta_data = """>robot

AATTG

>dog

GGGCA

>mouse

AAGAC

>rat

GTTGT

>cat

GAGGC

>ostrich

GTGTC

>elephant

AATTC"""

\# Parse the tree

tree = Phylo.read(StringIO(newick_tree), "newick")

\# Parse the FASTA sequences

sequences = {}

for record in SeqIO.parse(StringIO(fasta_data), "fasta"):

    sequences[record.id] = str(record.seq)

\# Function to find reverse substitutions

def find_reversing_substitutions(tree, sequences):

    # Recursively explore the tree

    reversing_substitutions = []

    def traverse(node):

        if node.is_terminal():

            return sequences[node.name]

        # Assume binary tree, get left and right children

        left_seq = traverse(node.clades[0])

        right_seq = traverse(node.clades[1])

        parent_seq = sequences[node.name]

        for i in range(len(parent_seq)):

            if left_seq[i] != parent_seq[i] and right_seq[i] != parent_seq[i]:

                # Check for the reversing condition

                if left_seq[i] == right_seq[i] == parent_seq[i]:

                    continue

                if left_seq[i] == parent_seq[i] and right_seq[i] != left_seq[i]:

                    reversing_substitutions.append((node.clades[1].name, node.clades[0].name, i, f"{parent_seq[i]}->{right_seq[i]}->{left_seq[i]}"))

                if right_seq[i] == parent_seq[i] and left_seq[i] != right_seq[i]:

                    reversing_substitutions.append((node.clades[0].name, node.clades[1].name, i, f"{parent_seq[i]}->{left_seq[i]}->{right_seq[i]}"))

        return parent_seq

    traverse(tree.clade)

    return reversing_substitutions

\# Find and print the reversing substitutions

reversing_substitutions = find_reversing_substitutions(tree, sequences)

for rs in reversing_substitutions:

    species_change, position, substitution = rs[0:2], rs[2], rs[3]

    print(f"{species_change}: position {position}, {substitution}")

\# Expected output is printed based on found reversing substitutions

```

This code utilizes Biopython's `Phylo` module to parse the Newick tree and the `SeqIO` module for parsing the FASTA data. The function `find_reversing_substitutions` uses a recursive tree traversal to identify and output reversing substitutions. Adjust the implementation as necessary to fit the logic exactly based on reversing conditions in your dataset. The output should be consistent with identifying the reversing substitutions in the described format.

To determine the reversing substitutions based on the given tree and sequences, we need to manually trace each position across the tree's paths as detailed in the previous explanation. Let's go through this step-by-step to find the reversing substitutions:

\### Given Data:

- **Newick Tree:**

  ```

  (((ostrich,cat)rat,mouse)dog,elephant)robot;

  ```

- **Sequences:**

  ```

  robot:    AATTG

  dog:      GGGCA

  mouse:    AAGAC

  rat:      GTTGT

  cat:      GAGGC

  ostrich:  GTGTC

  elephant: AATTC

  ```

\### Approach:

1. **Tree Structure**: Visual representation of the binary tree structure:

   ```

       robot

       /      \

    dog    elephant

    /   \

  mouse

   /  \

rat

   /  \

ostrich  cat

   ```

2. **Analyzing Each Position**:

   - Traverse the tree from the root to leaves.

   - At each node, compare the symbol between parent-child.

3. **Identifying Reversing Substitutions**:

Let's examine each position:

\#### Position 0:

- **robot**: A

- **dog**: G

- **elephant**: A (matches with robot)

**Reversion from `elephant` to `robot` does not apply, since `dog` is not the original.**

\#### Position 1:

- **robot**: A

- **dog**: G

- **elephant**: A

**Reversion from `elephant` to `robot` applies, since `elephant`: A `->` dog: G `->` robot: A**

\#### Position 2:

- **robot**: T

- **dog**: G (matches none)

**No reversions found.**

\#### Position 3:

- **robot**: T

- **dog**: C (matches none)

**No reversions found.**

\#### Position 4:

- **robot**: G

- **dog**: A (matches none)

**No reversions found.**

\### Conclusion:

The reversing substitution found is:

- Between **elephant** and **robot** at **position 1**: A -> G -> A

**Final Output:**

```

(elephant, robot): position 1, A->G->A

```

This result indicates a substitution at position 1 starting with A in `elephant`, changing to G in `dog`, and reverting back to A in `robot`.

**Analysis:** incorrect, only gave 1 and it was incorrect

##############################################################################

**Linguistic Complexity of a Genome**

Given: A DNA string s of length at most 100 kbp.

Return: The linguistic complexity lc(s).

Sample Dataset

ATTTGGATT

The linguistic complexity of a string s is defined as the ratio of the number of distinct substrings (of all possible lengths) to the maximum possible number of distinct substrings.

**Steps to Calculate Linguistic Complexity**

- **Count Distinct Substrings**:
  - Identify all distinct substrings of s. For a string of length n, consider all substrings of length 1 to n.
- **Maximum Possible Distinct Substrings**:
  - The maximum possible number of distinct substrings for a string of length n is calculated as:
    Maximum distinct substrings=∑k=1nk=n×(n+1)2
  - This formula counts all possible substrings without considering duplicates.
- **Compute Linguistic Complexity**:
  - The linguistic complexity lc(s) is calculated as:
    lc(s)=Number of distinct substringsMaximum possible distinct substrings

**Implementation in Python**

Using Python, we can efficiently count distinct substrings by employing a set data structure to collect the substrings:

def calculate_linguistic_complexity(s):

    n = len(s)

    distinct_substrings = set()

    # Generate all possible substrings

    for length in range(1, n + 1):

        for start in range(n - length + 1):

            substr = s[start:start + length]

            distinct_substrings.add(substr)

    num_distinct_substrings = len(distinct_substrings)

    max_possible_substrings = (n * (n + 1)) // 2

    # Linguistic complexity

    lc = num_distinct_substrings / max_possible_substrings

    return lc

\# Sample Input

s = "ATTTGGATT"

\# Calculate linguistic complexity

linguistic_complexity = calculate_linguistic_complexity(s)

print(f"Linguistic Complexity: {linguistic_complexity}")

**Example Calculation for the Sample Dataset**

For the sample dataset s="ATTTGGATT":

- **Distinct Substrings**: Determine all unique substrings of each length:
  - Length 1: A, T, G
  - Length 2: AT, TT, TG, GG, GA
  - Length 3: ATT, TTT, TGG, GGA, GAT, TGA,
  - ...continue for lengths up to 8...
- **Count**: Total number of distinct substrings.
- **Maximum Possible**:
  - 9×102=45
- **Linguistic Complexity**:
  - Assume |Distinct substrings|=36
  - Linguistic Complexity = 3645=0.8

The output will be a number between 0 and 1 representing the linguistic complexity of the string based on these calculations. This gives an insight into how well s covers the possible substrings it can form.

what is the output

To provide the output for the linguistic complexity of the sample dataset s="ATTTGGATT", we will calculate both the number of distinct substrings and the maximum possible number of distinct substrings, and then compute the linguistic complexity.

**Step-by-Step Calculation**

**1. String Length:**

- Length of s, n=9.

**2. Maximum Possible Distinct Substrings:**

- Formula: n×(n+1)2
- Calculation: 9×102=45

**3. Distinct Substrings:**

- We can enumerate all distinct substrings of s.
- All single characters: A, T, G
- Substrings of length 2: AT, TT, TG, GG, GA
- Substrings of length 3: ATT, TTG, TGG, GGA, GAT
- Substrings of length 4: ATTT, TTGG, TGGA, GGAT
- Substrings of length 5: ATTTG, TTGGA, TGGAT
- Substrings of length 6: ATTTGG, TTGGAT
- Substrings of length 7: ATTTGGA, TTGGATT
- Substrings of length 8: ATTTGGAT
- Substrings of length 9: ATTTGGATT

After compiling these, count the number of unique substrings, which is the number of elements in the list.

**4. Distinct Substring Calculation:**

Let's enumerate them properly:

- Given substrings generation, each substring is unique in its context through compiled length.
- Counting distinct elements gives us approximately 36 unique distinct formations for substrings with diverse repeating cycles from isolated characters.

**5. Linguistic Complexity Calculation:**

Let's assume that through improved step verification, you correctly compute:

- **Number of distinct substrings:** 36 (this assumption is based on typical enumeration but ensures diligence in improbability or set calculation)
  Using reviewed enumeration, compute:
- **Linguistic Complexity:**
  lc(s)=3645=0.8

**Output:**

Linguistic Complexity: 0.8

This output presents the linguistic complexity, indicating the ratio of possible distinct substrings actually found within the string relative to the total possible, assuming systematic calculation on detailed streamlined processing. If enumerating or scripting reminders better helps operational accuracy, ensure calculated design facilitates customized count precision.

Analysis: correct

##############################################################################

**Llama-3 Examples**

**Given:** A list containing n taxa (n≤2000) and two unrooted binary trees T1 and T2 on the given taxa. Both T1 and T2 are given in [Newick format](https://rosalind.info/glossary/newick-format/).

**Return:** The quartet distance dq(T1,T2).

**Sample Dataset**

**A B C D E**

**(A,C,((B,D),E));**

**(C,(B,D),(A,E));**

To provide the sample output, we first need to conduct a theoretical calculation of the quartet distance between the two given Newick format trees. Let's analyze the trees:

\### Given Trees:

1. **Tree T1**: `(A,C,((B,D),E));`

    - Implies a structure where B and D are more closely related with E than with A and C.

2. **Tree T2**: `(C,(B,D),(A,E));`

    - Implies a structure where B and D are grouped together, different from the specific grouping in T1, but implication differs for A and E.

\### Basic Quartet Counting:

- **Extract Quartets**:

  - From **T1**, the quartets are derived from the specific subtree relations:

    - B and D are together separated by E from both A and C, inferring possible quartets like AB|CD, AC|BD, etc.

  - From **T2**, the quartets are based on how (B, D) are separated with A and E from C.

Given the limited data visibility in character format, let’s assume these trees as minimal:

- Calculate number of total quartets possible among five taxa. From combinatorial rules, \( \binom{n}{4} \) quartets are possible:

  - For 5 taxa, this is \( \binom{5}{4} = 5 \).

\### Realize and Compare:

- Compare each possible quartet inferred from T1 to those from T2:

  - Differences and overlaps in quartets give us \( q(T1) \), \( q(T2) \), and common quartets \( q(T1,T2) \).

\### Calculate Quartet Distance (Theory):

1. Assume distinct sets and calculate:

  - \( q(T1) = 5 \), \( q(T2) = 5 \), because each covers possible quartets among 5.

  - Common quartets (say) \( q(T1, T2) = 3 \).

2. Plug into formula:

   \[ dq(T1, T2) = 5 + 5 - 2 \times 3 = 10 - 6 = 4. \]

Thus, based on theoretical inference and surmised layout, the quartet distance for this pair of tetra-taxon trees is \( \boxed{4} \). This conclusion considers assumptions made since interpreting full exactness requires more in-depth analysis or parsed execution.

**Analysis: correct**

---------------------------------------------------------------------------------------------------------------------

Given: A DNA string s of length at most 1 kbp.

Return: A list containing all maximal repeats of s having length at least 20.

Sample Dataset

TAGAGATAGAATGGGTCCAGAGTTTTGTAATTTCCATGGGTCCAGAGTTTTGTAATTTATTATATAGAGATAGAATGGGTCCAGAGTTTTGTAATTTCCATGGGTCCAGAGTTTTGTAATTTAT

To find all maximal repeats of a given DNA string s with length at least 20, we can utilize suffix trees or suffix arrays with LCP (Longest Common Prefix) arrays. These structures allow us to efficiently identify repeated substrings within a string. Here’s a step-by-step approach you can use manually or programmatically to solve such a problem:

**Steps to Identify Maximal Repeats:**

- **Construct the Suffix Array and LCP Array:**
  - **Suffix Array:** A sorted array of all suffixes of the string. Each suffix is represented by its starting index.
  - **LCP Array:** The LCP array is an array of the longest common prefix lengths between each pair of consecutive suffixes in the suffix array.
- **Identify Repeats Using LCP:**
  - Traverse the LCP array to identify lengths of repeated substrings.
  - A maximal repeat corresponds to an entry in the LCP array (i.e., a common prefix in the suffix array) that cannot be extended to the left or right.
- **Filter and Determine Maximal Repeats:**
  - For each candidate repeat of length l in the LCP array, verify that this substring cannot be extended.
  - Filter the list to include only repeats of length 20 or more.
- **Return the Maximal Repeats:**
  - Compile a list of these maximal repeats and return them.

Unfortunately, this generic explanation doesn't directly solve the sample dataset, but here's a manual example for clarity:

Given the DNA string:

TAGAGATAGAATGGGTCCAGAGTTTTGTAATTTCCATGGGTCCAGAGTTTTGTAATTTATTATATAGAGATAGAATGGGTCCAGAGTTTTGTAATTTCCATGGGTCCAGAGTTTTGTAATTTAT

Upon building suffix and LCP arrays, look for entries in the LCP array with values 20 or more, check those substrings in the original sequence, and ensure they cannot be extended:

Assume maximal repeats might be identified after examination as:

- "AGTGGGTCCAGAGTTTTGTA" (21 characters)
- "GATAGAATGGGTCCAGAGT" (20 characters)

**Note:** Because maximal repeat identification algorithms are computational and involve significant suffix array manipulations, a full step-through example might often rely on algorithm implementation utilizing the steps above.

If you prefer a coding solution, Python libraries such as strAlgo or implementing manually using Python's sorted() for suffixes can suffice for a small string like 1 kbp.

**Analysis: incorrect**, alignment issue

##############################################################################

**Given:** A positive integer n (4≤n≤5000), followed by an [unrooted binary tree](https://rosalind.info/glossary/unrooted-binary-tree/) T in [Newick format](https://rosalind.info/glossary/newick-format/) on n taxa.

**Return:** The value of q(T) [modulo](https://rosalind.info/glossary/modular-arithmetic/) 1,000,000.

A quartet AB∣CD is consistent with a binary tree T if the quartet can be inferred from one of the splits of T (see “Quartets” for a description of inferring quartets from splits).

Let q(T) denote the total number of quartets that are consistent with T.

Given: A positive integer n (4≤n≤5000), followed by an unrooted binary tree T in Newick format on n taxa.

Return: The value of q(T) modulo 1,000,000.

Sample Dataset

6

(lobster,(cat,dog),(caterpillar,(elephant,mouse)));

The problem is to calculate the total number of quartets that are consistent with a given unrooted binary tree T.

To solve this problem, we can use the following approach:

- Parse the Newick format tree and create a representation of the tree in memory.
- Calculate the total number of quartets consistent with the tree by counting the number of ways to choose four taxa and then checking if the quartet can be inferred from one of the splits of the tree.
- Return the result modulo 1,000,000.

Here is a Python solution using the dendropy library to parse the Newick format tree:

import dendropy

from math import comb

def count_quartets(tree):

    n = tree.taxon_namespace.count()

    return (n * (n-1) * (n-2) * (n-3)) // 24

def solve(n, tree_string):

    tree = dendropy.Tree.get(string=tree_string, schema='newick')

    result = count_quartets(tree) % 1000000

    return result

n = 6

tree_string = '(lobster,(cat,dog),(caterpillar,(elephant,mouse)));'

print(solve(n, tree_string))

However, this solution assumes that the tree is a binary tree and that all quartets can be inferred from one of the splits of the tree. If this is not the case, a more complex approach would be needed to calculate the total number of quartets consistent with the tree.

Also, note that the count_quartets function calculates the total number of quartets that can be formed with n taxa, which is (n choose 4). This assumes that all quartets can be inferred from one of the splits of the tree.

In a more general case, we would need to iterate over all internal edges in the tree, and for each edge, count the number of taxa on each side of the edge. Then, we would multiply the number of ways to choose two taxa from each side, and add this to the total count of quartets.

Here is a more general Python solution:

import dendropy

def count_quartets(tree):

    result = 0

    for edge in tree.postorder_edge_iter():

        if edge.is_internal():

            left_taxa = set()

            right_taxa = set()

            for node in edge.head_node_iter():

                for leaf in node.leaf_iter():

                    left_taxa.add(leaf.taxon.label)

            for node in edge.tail_node_iter():

                for leaf in node.leaf_iter():

                    right_taxa.add(leaf.taxon.label)

            result += len(left_taxa) * (len(left_taxa) - 1) // 2 * len(right_taxa) * (len(right_taxa) - 1) // 2

    return result % 1000000

def solve(n, tree_string):

    tree = dendropy.Tree.get(string=tree_string, schema='newick')

    return count_quartets(tree)

n = 6

tree_string = '(lobster,(cat,dog),(caterpillar,(elephant,mouse)));'

print(solve(n, tree_string))

This solution iterates over all internal edges in the tree, counts the number of taxa on each side of the edge, and multiplies the number of ways to choose two taxa from each side. This gives the total number of quartets that can be inferred from the tree.

provide the sample output

The sample output for the given sample dataset is:

15

This means that there are 15 quartets that are consistent with the given unrooted binary tree T.

Here is the sample output with the code executed:

def solve(n, tree_string):

    import dendropy

    tree = dendropy.Tree.get(string=tree_string, schema='newick')

    result = 0

    for edge in tree.postorder_edge_iter():

        if edge.is_internal():

            left_taxa = set()

            right_taxa = set()

            for node in edge.head_node_iter():

                for leaf in node.leaf_iter():

                    left_taxa.add(leaf.taxon.label)

            for node in edge.tail_node_iter():

                for leaf in node.leaf_iter():

                    right_taxa.add(leaf.taxon.label)

            result += len(left_taxa) * (len(left_taxa) - 1) // 2 * len(right_taxa) * (len(right_taxa) - 1) // 2

    return result % 1000000

n = 6

tree_string = '(lobster,(cat,dog),(caterpillar,(elephant,mouse)));'

print(solve(n, tree_string))

Output:

15

**Analysis: correct**

---------------------------------------------------------------------------------------------------------------------

Given: A DNA string s of length at most 1kbp.

Return: The substrings of s∗ encoding the edges of the suffix tree for s. You may list these substrings in any order.

Sample Dataset

ATAAATG$

Here's a Python solution to this problem using the Ukkonen's algorithm for constructing a suffix tree:

class Node:

    def __init__(self, label):

        self.label = label

        self.children = {}

class SuffixTree:

    def __init__(self, string):

        self.root = Node("root")

        self.string = string + "$"

        self.build_tree()

    def build_tree(self):

        for i in range(len(self.string)):

            current = self.root

            for j in range(i, len(self.string)):

                if self.string[j] not in current.children:

                    current.children[self.string[j]] = Node(self.string[i:j+1])

                current = current.children[self.string[j]]

    def get_edges(self):

        edges = []

        self._get_edges(self.root, edges)

        return edges

    def _get_edges(self, node, edges):

        for child in node.children.values():

            edges.append(child.label)

            self._get_edges(child, edges)

def main():

    # Sample dataset

    data = "ATAAATG$"

    # Build the suffix tree

    tree = SuffixTree(data)

    # Get the edges of the suffix tree

    edges = tree.get_edges()

    # Print the edges

    for edge in edges:

        print(edge)

if __name__ == "__main__":

    main()

This code defines a Node class to represent the nodes of the suffix tree and a SuffixTreeclass to represent the suffix tree itself. The build_tree method constructs the suffix tree using Ukkonen's algorithm, and the get_edges method returns a list of the substrings labeling the edges of the suffix tree.

When you run this code with the sample dataset "ATAAATG$", it will print the substrings labeling the edges of the suffix tree. The output will be:

A

T

AA

ATAAATG$

A

AT

AAATG$

G$

Note that the order of the edges in the output may vary depending on the implementation of the get_edges method.

**Analysis:** incorrect, provided only 8 substrings instead of 12
